# Retraction: Cord blood stem cell-mediated induction of apoptosis in glioma downregulates X-linked inhibitor of apoptosis protein (XIAP)

**DOI:** 10.1371/journal.pone.0327907

**Published:** 2025-07-11

**Authors:** The PLOS One Editors

Following the publication of this article [1] concerns were raised about the results presented in Figs 1, 3–6, and S2–S3. Specifically,

- The following panels appear similar:
  - Fig 1D Akt and Fig 1E pAkt^Ser473^ when flipped vertically
  - Fig 1D GAPDH, and Fig 1E XIAP and Fig 1E GAPDH when rotated 180°
  - Fig 3A Bax (left), Fig 3A Bad (left) when flipped vertically, and Fig 4C pAkt^S473^ when flipped horizontally
  - Fig 3A Bcl-2 (left) and Fig 3A Bcl-2 (right)
  - Fig 3A GAPDH (left) and Fig 3A GAPDH (right)
  - Fig 4C GAPDH (top) of this article [1] and Fig 6E GAPDH (left) of [2, corrected in 3]
  - Fig 5D XIAP of this article [1] and Fig 5A XIAP of [4, retracted in 5]
  - Fig 5D Akt of this article [1], Fig 5A Akt of [4, retracted in 5], and Fig 6A FAK of [6]
  - Fig 5D GAPDH and Fig S3 U251 GAPDH of this article [1], Fig 5A GAPDH of [4, retracted in 5], Fig 5B GAPDH of [4, retracted in 5], Fig S3 GAPDH of [7] and Fig 7C GAPDH of [8, retracted in 9]
  - Fig 5F XIAP of this article [1], Fig 5C XIAP of [4, retracted in 5], Fig 5C PDGFR of [4, retracted in 5], and Fig 6G VEGFR1 of [6]
  - Fig 5F β-actin of this article [1] and Fig 5C β-actin of [4, retracted in 5]
  - Fig 6B TNF-α of this article [1] and Fig 7I STAT-3 of [8, retracted in 9]
  - Fig S3 5310 XIAP and Fig S3 5310 Akt
- The following panels appear to partially overlap:
  - Fig S2 U251 XIAP (lanes 1-2) and Fig S2 5310 XIAP (lanes 1-2) when flipped horizontally
  - Fig S2 SNB19 Bax and Fig S3 5310 TRAIL (lanes 2-4) when flipped vertically
  - Fig S2 U251 Bax (lanes 1-2) and Fig S3 5310 TRAIL (lanes 1-2) when rotated 180°
  - Fig S3 5310 GAPDH (lanes 1-4) of this article [1] and Fig 7G GAPDH (lanes 3-6) of [8, retracted in 9]
  - Fig S3 5310 GAPDH (lanes 1-3) of this article [1] and Fig 1B GAPDH (lanes 3-5) of [4, retracted in 5]

The authors did not respond to the journal’s communications.

In light of the above concerns that question the reliability and integrity of these results, the *PLOS One* Editors retract this article.

All authors either did not respond directly or could not be reached.
